# EO771, the first luminal B mammary cancer cell line from C57BL/6 mice

**DOI:** 10.1186/s12935-020-01418-1

**Published:** 2020-07-20

**Authors:** Augustin Le Naour, Yvonne Koffi, Mariane Diab, Delphine Le Guennec, Stéphanie Rougé, Sahar Aldekwer, Nicolas Goncalves-Mendes, Jérémie Talvas, Marie-Chantal Farges, Florence Caldefie-Chezet, Marie-Paule Vasson, Adrien Rossary

**Affiliations:** 1 Human Nutrition Unit, ECREIN team, UMR 1019, University of Clermont Auvergne, INRAE, CRNH-Auvergne, TSA 50400, 28 place Henri Dunant, 63000 Clermont-Ferrand Cedex 1, France; 2 Department of Nutrition, Gabriel Montpied University Hospital, Jean Perrin Cancer Centre, 58 rue Montalembert, 63011 Clermont-Ferrand, France

**Keywords:** Antineoplastic agents, Hormonal, Breast neoplasms, Mice, inbred C57BL, Receptors, estrogen, Tamoxifen

## Abstract

**Background:**

Despite decades of therapeutic trials, effective diagnosis, many drugs available and numerous studies on breast cancer, it remains the deadliest cancer in women. In order to choose the most appropriate treatment and to understand the prognosis of the patients, breast cancer is divided into different subtypes using a molecular classification. Just as there remains a need to discover new effective therapies, models to test them are also required.

**Methods:**

The EO771 (also named E0771 or EO 771) murine mammary cancer cell line was originally isolated from a spontaneous tumour in C57BL/6 mouse. Although frequently used, this cell line remains poorly characterized. Therefore, the EO771 phenotype was investigated. The phenotype was compared to that of MCF-7 cells, known to be of luminal A subtype and to express estrogen receptors, as well as MDA-MB-231 cells, which are triple negative. Their sensitivity to hormonal treatment was evaluated by viability tests.

**Results:**

The EO771 were estrogen receptor α negative, estrogen receptor β positive, progesterone receptor positive and ErbB2 positive. This phenotype was associated with a sensitivity to anti-estrogen treatments such as tamoxifen, 4-hydroxy-tamoxifen, endoxifen and fulvestrant.

**Conclusions:**

On account of the numerous results published with the EO771 cell line, it is important to know its classification, to facilitate comparisons with corresponding types of tumours in patients. Transcriptomic and protein analysis of the EO771 cell line classified it within the luminal B subtype. Luminal B cancers correspond to one of the subtypes most frequently encountered in patients and associated with a poor prognosis.

## Background

Breast cancer is the most common, deadliest female cancer [1]. Breast cancer is a heterogeneous disease whose prognosis and treatment depend on the molecular classification [2, 3] (Table 1). The expression of hormone receptors such as estrogen receptors alpha (ERα) and beta (ERβ) as well as progesterone receptors (PR) are used routinely by anatomo-pathologists to classify the type of tumour. To complete this, the expression of Human Epidermal growth factor Receptor 2 (HER2 also named ERBB2) is investigated. Thus, different tumour subtypes are described: luminal A characterized by expression of hormone receptors ER + and/or PR + , absence of overexpression of *ERBB*2 [4] gene whereas luminal B cancers showed lower expression of ER and PR, but frequently associated with an increased expression of growth factor receptor genes such as *HER2* [5]. The basal-like or triple negative breast cancer do not express any of these markers: ER-, PR-, ERBB2- [4].

**Table 1:** “Intrinsic” subtypes of breast cancer

“Intrinsic” subtypes of breast cancer	Estrogen receptors (ER)	Progesterone receptors (PR)	Human epidermal growth factor receptor 2 (HER2)	Other major characteristics
Luminal A	ER +	PR ±	HER2−	
Luminal B	ER +	PR ±	HER2 +	
Triple negative or basal-like	ER−	PR−	HER2−	
Triple negative claudin low	ER−	PR−	HER2−	Claudin-3, claudin-4 and claudin-7 low
HER2 +	ER−	PR−	HER2 +	

It is, therefore, important to know what type of tumour the research is carried out in order to be able to transpose the results to patients. Preclinical models are needed in order to better understand the development of the cancer and to test new therapies to improve their management. Preclinical studies require working on animal models that can mimic human pathology. Thus, orthotopic syngeneic models have the advantage of having the whole breast microenvironment and, therefore, being able to mimic the human pathology as well as possible. To carry out in vivo experiments, the murine model is frequently used. The C57BL/6 strain is the most used, the genome of which has already been sequenced first [6] and of which there are many transgenic strains (knockout, knockdown, overexpression, etc). EO771 cells are from C57BL/6 mice. Thus, a mouse syngeneic model of mammary adenocarcinoma can be used by orthotopic injection of EO771 (also named E0771 or EO 771) cells into C57BL/6 mice. These cells could be widely used for preclinical studies.

This model facilitates generation of an immunocompetent model of breast cancer in vivo [7, 8]. However, it is necessary to know their subtype classification in order to be able to establish a parallel between the results obtained with this line and the patients who would be potentially involved. Nevertheless, this type of mammary tumour remains poorly characterized and the results are divergent concerning its classification. To date, among the 100 publications studying EO771 cells, 10 articles consider them as triple negative and 19 as ERα + . However, only 3 articles analysed the expression of ERα [9–11]. Johnstone et al. investigated ERα by immunohistochemistry in EO771 cells but considered these cells as ERα- because the expression of this receptor was only found in the cytoplasm and not in the nuclear compartment [9]. Thus, some researchers prefer to mention the unclear status of this cell line [12, 13]. No model of breast cancer derived from C57BL/6 mice is currently well characterized in terms of their molecular classification. Thus, it is important to better characterize this syngeneic EO771 mammary adenocarcinoma cell line in order to determine the type of tumour that is closest to those found in patients.

This study first characterized the EO771 cell line concerning its classification and evaluated its sensitivity to anti-estrogen therapy. The identification of signalling pathways, activated after anti-estrogen therapy, was also investigated. Thus, the results shown that EO771 cells displayed a luminal B phenotype, characterized by a phenotype: ERα-, ERβ + , PR + and ErbB2 + . This cell line was sensitive to anti-estrogen drugs such as tamoxifen, which induced an activation of pro-apoptotic signalling pathway as c-Jun NH2-terminal kinase (JNK) and p38 mitogen-activated protein kinase (MAPK) families.

Thus, this article shows for the first time that the EO771 line belongs to the luminal B subtype. Our studies are, therefore, important for transposing the results obtained on this tumour line to patients with luminal B cancer, which corresponds to one of the subtypes most frequently encountered in patients and one which is associated with a poor prognosis.

## Materials and methods

### Mammary adenocarcinoma cell lines

Mouse mammary cancer cell line EO771 (CH3 BioSystems, Amherst, NY), human triple negative breast cancer cell line MDA-MB-231 (American Type Culture Collection (ATCC), Molsheim, France) and human luminal A breast cancer cell line MCF-7 (ATCC) were grown in DMEM supplemented with foetal calf serum (10%), L-Glutamine (1%) and penicillin / streptomycin (1%) at 37 °C in 5% CO_2_.

### Cell viability assay

EO771, MCF-7 and MDA-MB-231 cells were plated at a density of 2 × 10^3^ cells in 96-well plates in a complete medium. Cultures were at 37 °C in a humidified atmosphere with 5% CO_2_. Two days later, cells were treated by a range of tamoxifen, 4-hydroxy-tamoxifen (4-OH-tamoxifen), endoxifen or fulvestrant (1–25 μM). A supplementation with estradiol (low: 1.5 ng/mL; high: 225 ng/mL), leptin (low: 10 ng/mL; high: 100 ng/mL) or dimethyl sulfoxide (DMSO) (0.25%) were also carried out. Estradiol concentrations were chosen to be close to the estradiol concentrations measured in mice bearing EO771 tumours observed in our laboratory (data not shown) (low: 1.5 ng/mL; high: 225 ng/mL). Leptin was used at 10 ng/mL (low concentration) and 100 ng/mL (high concentration), close to the physiological conditions [14] and the final concentration in DMSO was 0.25% in all wells. After 48, 72, and 96 h, cells were washed with phosphate-buffered saline (PBS) and loaded with 100 μL of a 25 μg/mL solution of resazurin in DMEM for each well. Resazurin is an indicator of viable cells. As with an MTT test, cells that have an active metabolism can reduce resazurin to resorufin, which is pink and fluorescent. The amount of resorufin produced is proportional to the number of viable cells. The amount of resorufin produced is evaluated by fluorescence. The plates were incubated for 2 h at 37 °C in a humidified atmosphere containing 5% CO_2_. Fluorescence intensity was then measured on an automated 96-well plate reader (Fluoroskan Ascent FL, Thermo Fisher Scientific, Wilmington, DE, USA) using an excitation wavelength of 530 nm and an emission wavelength of 590 nm. Under these conditions, fluorescence was proportional to the number of living cells in the well [15].

### EO771 cell treatment by anti-estrogen, estradiol and leptin

EO771 cells were plated at a density of 4.5 × 10^5^ cells in 75 cm^2^ flasks in a complete medium. Cultures were at 37 °C in a humidified atmosphere with 5% CO_2_. After 48 h, they were treated by the tamoxifen IC50 (14 µM) previously determined. Estradiol (low: 1.5 ng/mL; high: 225 ng/mL), leptin (low: 10 ng/mL; high: 100 ng/mL) and DMSO (final concentration of 0.25% in all flasks) were also added. After 72 h of treatments, cells were harvested and protein and RNA were extracted.

### RNA extraction

Total RNA from EO771 cells was extracted by TRIzol® reagent (Invitrogen, Saint Aubin, France) according to the manufacturer’s protocol, and quantified using a NanoDrop spectrophotometer (NanoDrop®2000, Thermo Scientific, Waltham, MA, USA). Reverse transcription was performed in a thermocycler (Mastercycler® gradient; Eppendorf, Montesson, France) on 1 µg of total RNA for each condition using a high-capacity cDNA reverse transcription kit (Applied Biosystems, Saint Aubin, France) with random hexamer pdN6 primers.

### Quantitative real-time Polymerase Chain Reaction (q-PCR)

q-PCR was performed using SYBR®Green reagents according to the manufacturer’s instructions on a StepOne system (Applied Biosystems). Each condition was assayed in triplicate. Relative quantification was obtained by the comparative Ct method, based on the formula 2^−∆∆Ct^. As the GAPDH mRNA levels were consistent across EO771, MCF7, and MDA-MB-231 cell lines (Additional file 1: Figure S1), expression levels were normalized to the housekeeping gene (GAPDH) for each time point. The reference used corresponds to the value obtained by untreated MCF-7. Sequences and fragment sizes of the human and mouse specific primers used are reported in Table 2.

**Table 2:** Primers used for q-PCR analysis

Gene	Species	Size (bp)	RefSeq	Forward primer	Reverse primer
Aromatase	Human	207	NM_000103.3	5′-TGGCAAGCTCTCCTCATCAA-3′	5′-TGTCAGGTCACCACGTTTCT-3′
Aromatase	Mouse	142	NM_007810.4	5′-GCTACGTGGATGTGTTGACC-3′	5′-TTGATGAGGAGAGCTTGCCA-3′
ERBB2	Human	172	NM_004448.3	5′-TGTGTGGGAGCTGATGACTT-3′	5′-TCTTGGCCGACATTCAGAGT-3′
Erbb2	Mouse	152	NM_001003817.1	5′-GGCTGCTGGACATTGATGAG-3′	5′-AAAGGTCATCAGCTCCCACA-3′
ERα	Human	265	NM_000125.3	5′-GTGCCTGGCTAGAGATCCTG-3′	5′-AGAGACTTCAGGGTGCTGGA-3′
Erα	Mouse	231	NM_007956.5	5′-ATTATGGGGTCTGGTCCTGC-3′	5′-CATCTCTCTGACGCTTGTGC-3′
ERβ	Human	173	NM_001040275.1	5′-AAGAAGATTCCCGGCTTTGT-3′	5′-TCTACGCATTTCCCCTCATC-3′
Erβ	Mouse	202	NM_207707.1	5′-CTTTGCTCCAGACCTCGTTC-3′	5′-AATCATGGCCTTCACACACA-3′
GAPDH	Human	149	NM_001289745.3	5′-TGACCACAGTCCATGCCATC-3′	5′-CAGCTCAGGGATGACCTTGC-3′
Gapdh	Mouse	109	NM_001289726.1	5′-ACCCCAGCAAGGACACTGAGCAAG-3′	5′-GGCCCCTCCTGTTATTATGGGGGT-3′
PR	Human	224	NM_001202474.3	5′-ATGGAAGGGCAGCACAACTA-3′	5′-AGGGCTTGGCTTTCATTTGG-3′
Pr	Mouse	196	NM_008829.2	5′-CAGCGCTTCTACCAACTCAC-3′	5′-TTTTGTGAAAGAGGAGCGGC-3′

### Western blot analysis

Protein extractions were performed by RIPA buffer (ThermoFisher Scientific) supplemented with protease (Thermo Scientific Halt Protease Inhibitor Cocktail) and phosphatase (Halt Phosphatase Inhibitor Cocktail) inhibitors. 15 μg of extracted proteins were separated by SDS-PAGE and revealed by antibodies directed against actin (1:1000, Cell Signaling Technology #8457), Glyceraldehyde-3-phosphate dehydrogenase (GAPDH) (1:1000, Cell Signaling Technology #5174), ERα (1:1000, Abcam #ab32063), ERβ (1:500, Abcam #ab3576), PR ( ab133526), ErbB2 (1:1000, Cell Signaling Technology #4290) and the use of HRP Goat Anti-Rabbit (IgG) secondary antibody (1:5000, Abcam #ab6721). Alignment of ER, PR and aromatase peptide sequence shows more than 80% of homology between mouse's and human's proteins. For example, PR shows 80% of homology between the total sequences of the two species. The antibody used is designed on the C terminal domain which presents 95% of homology.

### Quantification of signalling pathway protein

Using Multiplex Biomarker Immunoassays (cat. kit 48-680MAG and 48-681MA) according to the manufacturer’s instructions, both total and phosphorylated forms of signalling pathways (cAMP-response element binding protein (CREB), JNK, Nuclear factor-κB (NF-κB), p38 MAPK, Extracellular signal-regulated kinase (ERK)1/2, AKT, p70S6K, Signal Transducer and Activator of Transcription (STAT) 3 and STAT5) and Matrix metalloproteinase-3 (MMP3) were determined in EO771 cell line, with and without tamoxifen treatment at IC50 (14 µM) for 48 h. The mean fluorescence intensity (MFI) was detected by the Multiplex plate reader for all measurements (Luminex System, Bio-Rad Laboratories, Germany) using a Luminex system, Bio-Rad Laboratories software version 4.2.

### Statistics

For chemoresistance tests, RT-qPCRs and protein analysis, the comparison between groups was performed using a Wilcoxon-Mann Whitney test (independent non-parametric data). *p* values < 0.05 (*) indicate a significant difference. Statistical analyses were performed using GraphPad Prism5 (GraphPad Software, Inc., La Jolla, CA).

## Results

### EO771 cells have a luminal B mammary cancer-like phenotype

**Fig. 1:**
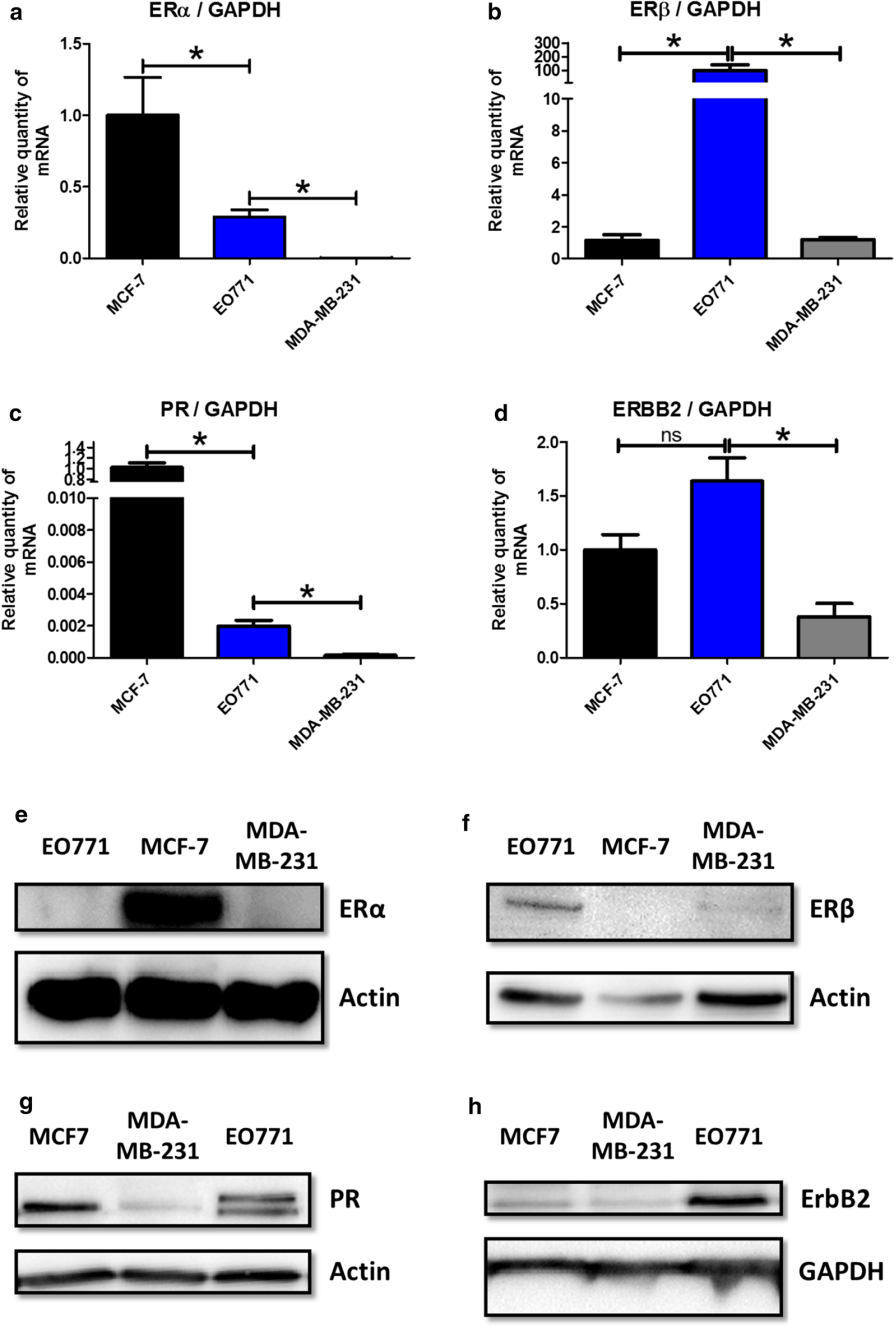
EO771 cells display a luminal B phenotype. **a**–**d** The relative expression of mRNA coding for ERα (**a**), ERβ (**b**), PR (**c**) and ERBB2 (**d**) was evaluated on MCF-7, MDA-MB-231 and EO771 cells. The values are normalized to the GAPDH gene expression. The data from MCF-7 were set to 1 and the relative quantity of mRNA is shown. *P* values of < 0.05 (*) using a Wilcoxon-Mann Whitney test indicate a significant difference. E–H: ERα (**e**), ERβ (**f**), PR (**g**) and ERBB2 (**h**) protein levels was assayed by western blot on MCF-7, MDA-MB-231 and EO771 cells (representative of 3 experiments) and normalized to the GAPDH or actin protein levels

The transcription of genes encoding ERα, ERβ, PR and ERBB2 was evaluated. EO771 cells were compared with human mammary tumour cell line MCF-7 considered to be ER + , PR + , HER2 − [16], i.e. luminal subtype A, as well as the human mammary tumour cell line MDA-MB-231 admitted as triple negative [17]. Although, the EO771 cells appeared to express ERs (Fig. 1a, b). They differed to MCF-7 in the transcription of the receptor subtypes. Indeed, in the MCF-7 cells, a strong transcription of ERα (Fig. 1a) but a small ERβ transcription was observed (Fig. 1b). In contrast, a significantly lower transcription of ERα was found in EO771 cells compared to MCF-7 (although its transcription was significantly greater than seen in MDA-MB-231 cells) (Fig. 1a). Whereas, the ERβ transcription was significantly greater than that observed in MCF-7 and MDA-MB-231 cells (Fig. 1b). EO771 cells expressed less PR than MCF-7 cells but this expression was superior to the triple negative cell line MDA-MB-231 (Fig. 1c). The 2 bands observed can be explained by the A and B isoforms of PR, expressed from a single gene [18]. Finally, the EO771 cells did not have an ERBB2 transcription significantly different from the MCF-7 cells, considered not over-expressing ERBB2, but significantly higher than the MDA-MB-231 cells (Fig. 1d). In view of these results, the EO771 line could be considered as ERα -, ERβ +, PR + and ERBB2 ±.

These results were confirmed by evaluating the protein expression of these receptors. Thus, strong ERα expression (Fig. 1e) was found for MCF-7 whereas it was undetectable for EO771 and MDA-MB-231 cell lines. In contrast, ERβ expression was higher in EO771 cells compared to MCF-7 and MDA-MB-231 cell lines (Fig. 1f). The expression of PR in EO771 cells was lower than that observed in MCF-7 (considered as PR +  [16]) but superior to MDA-MB-231 (considered as triple negative [17]) (Fig. 1g). Finally, concerning the ErbB2 receptor, the expression was greater in the EO771 cells compared with that of the MCF-7 and the MDA-MB-231 cells (Fig. 1h).

Finally, the results of the protein analysis confirm those of the gene transcription analysis allowing to classify the EO771 as luminal B subtype and more precisely ERα-, ERβ + , PR + and ErbB2 +.

### EO771 cells are sensitive to anti-estrogen treatments

The previous results have shown that EO771 cells expressed few ERα but in return, expressed more ERβ compared to MCF-7 cells. The impact of these receptor expression patterns on the sensitivity to anti-estrogenic treatment was evaluated. For that, the sensitivity of EO771 cells was tested to fulvestrant, a competitive estrogen receptor antagonist [19], and to tamoxifen [19, 20] as well as their active metabolites (endoxifen and 4-OH-tamoxifen), all of which are 3 competitive inhibitors of estrogen receptors with partial agonist activity. The ERα + cell line MCF-7, and the triple negative cell line MDA-MB-231, were used as positive and negative controls respectively. The human and murine estrogen receptors showed a great homology [21]. Interestingly, an in-silico study has shown that murine estrogen receptors interact with ligands in similar manner to the human receptor [22].

**Fig. 2:**
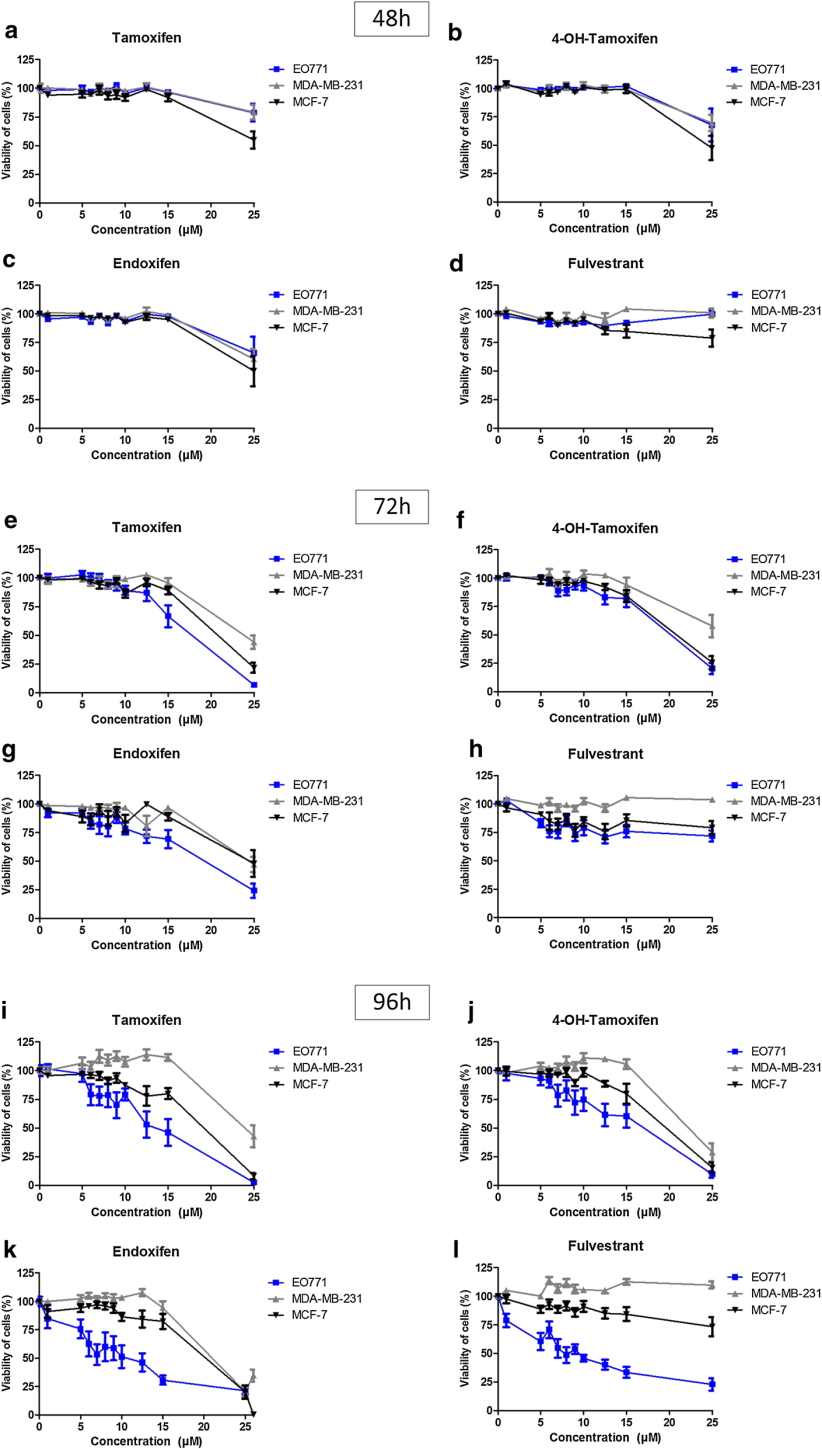
Anti-estrogenic treatments reduce EO771 cell viability. MCF-7, MDA-MB-231 and EO771 cells were treated with increasing tamoxifen (**a**, **e** and **i**), 4-OH-tamoxifen (**b**, **f** and **j**), endoxifen (**c**, **g** and **k**) and fulvestrant (**d**, **h** and **l**) concentrations for 48 h (**a**–**d**), 72 h (E–H) and 96 h (**i**–**l**). Cell viability was measured by fluorescence using resazurin solution. The untreated condition corresponds to a viability of 100%

Sensitivity to tamoxifen, 4-OH-tamoxifen, endoxifen and fulvestrant was almost zero in the first 48 h after treatment in all three cell lines (Fig. 2a–d). After 72 h of treatment, toxicity of tamoxifen treatments and its active metabolites (endoxifen and 4-OH-tamoxifen) was observed, but only at high dose (15 and 25 μM) on the three mammary cancer cell lines. The sensitivity of EO771 cells was slightly greater with tamoxifen (Fig. 2e) and endoxifen (Fig. 2g) compared to the two other cell lines. The sensitivity was comparable to that of MCF-7 for 4-OH-tamoxifen (Fig. 2f) but these two cell lines were nevertheless more sensitive to these drugs than MDA-MB-231. After 72 h of treatment with fulvestrant, the viability curves of EO771 and MCF-7 were comparable, showing a greater sensitivity of these two cell lines compared to MDA-MB-231 (Fig. 2h). Finally, after 96 h of treatment, the differences in sensitivity of the three tumour cell lines were more easily evaluable. As expected, the negative triple line MDA-MB-231 was the least sensitive line of the three, regardless of the hormone therapy used (tamoxifen, 4-OH-tamoxifen, endoxifen and fulvestrant) (Fig. 2i–l). More unexpectedly, a greater sensitivity of the EO771 cells to the four anti-estrogenic molecules was observed, compared to MCF-7 (Fig. 2i–l). Our work showed that the IC50 of tamoxifen on MCF-7 cells (close to 15 µM) is close to that observed by Zhang et al. [23]. Similarly, in agreement with our results, they observed that the MDA-MB-231 cells are resistant to tamoxifen. Thus, despite a low expression of ERα for EO771 cells compared to MCF-7 cells, it appears that the greater expression of ERβ allows a greater sensitivity to estrogen receptor-targeting treatments.

### The treatment with estradiol or leptin does not alter the phenotype nor the sensitivity to tamoxifen of EO771 cells

**Fig. 3:**
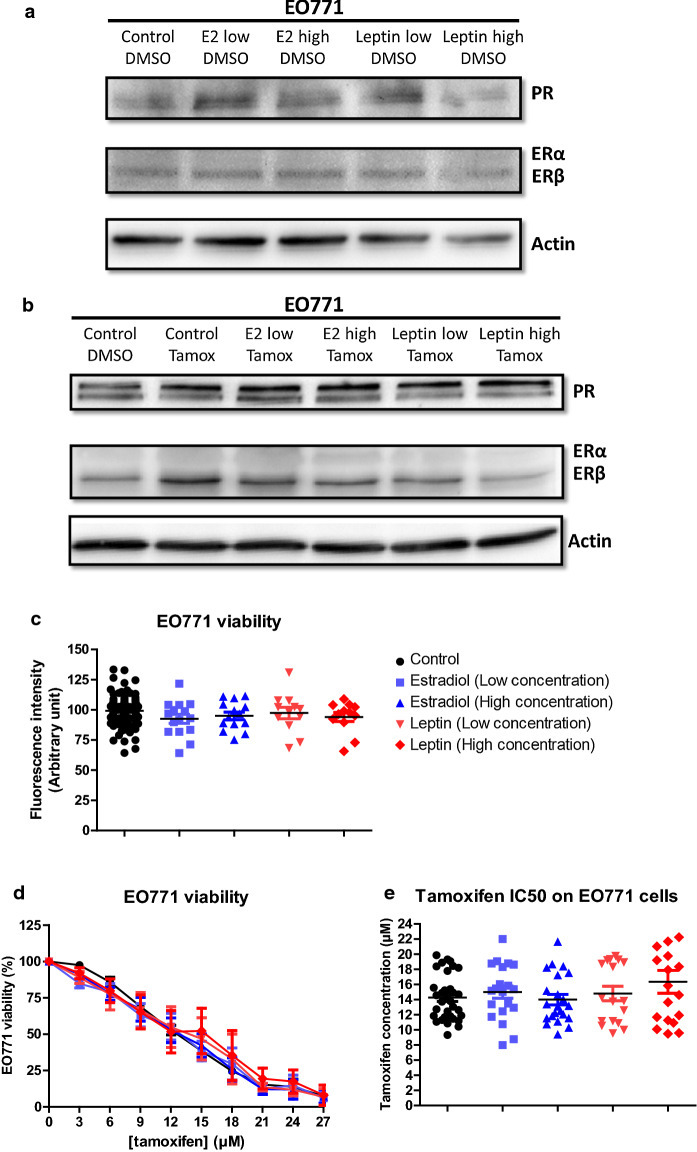
Estradiol and leptin do not influence EO771 cell phenotype and viability. **a**, **b** ERα, ERβ and PR protein levels was assayed by western blot on EO771 cells (representative of 3 experiments) and normalized to the actin protein levels in presence of estradiol (Low: 1.5 ng/mL; High: 225 ng/mL) or leptin (Low: 10 ng/mL; High: 100 ng/mL) in untreated conditions (DMSO) (**a**) or with tamoxifen (**b**). **c**, **d** Cell viability was measured by fluorescence using resazurin solution. EO771 cells were cultured in the presence of estradiol, leptin or vehicle (**c**) and EO771 cells were treated with increasing tamoxifen concentration (**d**). The untreated conditions corresponded to a viability of 100%. The tamoxifen IC50 corresponded to the tamoxifen concentration inducing 50% of EO771 viability (**e**)

The presence of estrogens could alter the sensitivity to anti-estrogenic agents in the EO771 cells, inducing competition in their binding to the ERs. Thus, the activity of tamoxifen could be modified in the presence of estradiol (E2). To evaluate this, the expression of hormone receptors in EO771, which would influence their sensitivity to tamoxifen, was investigated in the presence of estradiol in the culture medium. The effect of leptin was also evaluated because it decreases tamoxifen activity in MCF-7 cells [24] by increasing the nuclear expression of ERα [25] and leptin administration increased plasma estradiol levels [26, 27]. The ERα, ERβ, PR expression of the EO771 cells was only slightly modified in the presence of estradiol and leptin whether in the absence (Fig. 3a) or the presence of tamoxifen (Fig. 3b).

Before, in order to evaluate the sensitivity of EO771 cells in the presence of estradiol or leptin, the measure of the expression of the gene coding for aromatase (enzyme allowing the production of estrogens by androgen transformation) was studied in order to evaluate whether these cells were significantly able to produce estradiol. The gene coding for aromatase was very poorly transcribed in EO771 cells (Additional file 1: Figure S2). The study of protein expression by western blot did not detect aromatase in EO771 cells (data not shown). Thus, these cells did not seem capable of producing large amounts of estradiol.

In our experimental conditions, the addition of estradiol or leptin did not modify the growth of EO771 cells (Fig. 3c). Likewise, the sensitivity of EO771 cells to tamoxifen treatment was not modified with respect to the control condition (Fig. 3d). That was supported by the calculation of the concentrations of tamoxifen inhibiting 50% of cell viability (IC50). In fact, the IC50 values of tamoxifen for EO771 cells in the presence of estradiol or leptin were not significantly different compared to the control condition (Fig. 3e).

Thus, the presence of estradiol and leptin did not alter the luminal B phenotype nor the sensitivity to tamoxifen of EO771 cells.

### Tamoxifen activates signalling pathways in EO771 cells

Anti-estrogenic treatment leads to a cytotoxic effect on EO771 cells. However, the presence of estrogen has no effect on either proliferation, sensitivity to tamoxifen, or expression of hormone receptors. Thus, the response to tamoxifen observed in EO771 cells may be independent of estrogen receptors. Following this, the signalling pathways, which could be affected by tamoxifen treatment, were studied.

**Fig. 4:**
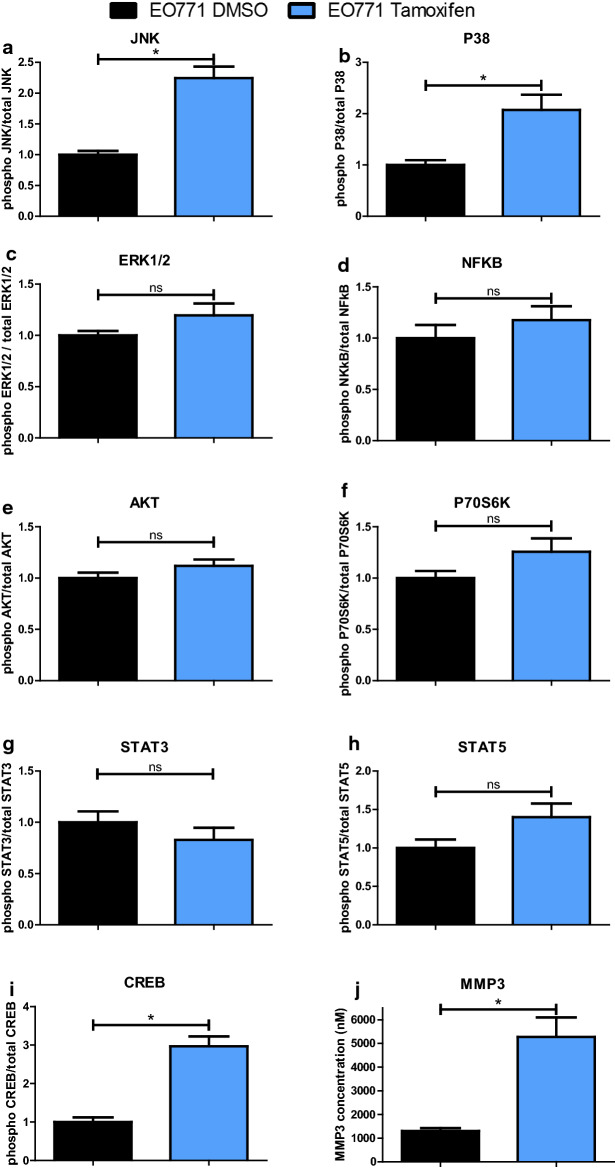
Tamoxifen modifies intracellular signalling pathways in EO771 cells. EO771 cells were treated by the tamoxifen IC50 (14 µM) or vehicle (DMSO) for 48 h. The expression of both total and phosphorylated forms of signalling pathway proteins (CREB, JNK, NFκB, p38, ERK1/2, AKT, p70S6K, STAT3 and STAT5) and MMP3 was analysed by measure of mean of fluorescence intensity (MFI) using a Luminex system. *P* values of < 0.05 (*) using a Wilcoxon-Mann Whitney test indicate a significant difference

The MAPK family pathway, which is a family of kinases that transduce signals from the cell membrane to the nucleus in response to numerous stimuli, was investigated. Classically, MAPKs are divided into three groups: ERK families that have an anti-apoptotic role, and the JNK and p38-MAPK families which are both associated to stress pathways leading to a pro-apoptotic effect [28]. The presence of tamoxifen led to the activation of pro-apoptotic pathways in EO771 cells such as the JNK and p38-MAPK pathways (Fig. 4a, b) without activating the ERK anti-apoptotic pathway (Fig. 4c). These results could suggest an activation of apoptosis under tamoxifen treatment.

The NF-κB pathway has been shown to be inhibited by tamoxifen [29]. Nevertheless, in our experiment, the presence of tamoxifen did not modify the NF-κB pathway activation in EO771 cells (Fig. 4d).

The PI3K/AKT pathway is a survival pathway leading to enhanced cell survival and cell cycle progression. In EO771 cells, the addition of tamoxifen did not cause any change in the activation of this pathway (Fig. 4e). Similarly, the p70S6K kinase, which is phosphorylated and activated by mTOR in mitogenic pathways downstream of PI3K/AKT, was not differently phosphorylated in tamoxifen-treated EO771 cells compared to the control condition (Fig. 4f).

Signalling pathways involving the STAT3 and STAT5 were also studied. Indeed, STAT5 assumes essential roles in proliferation, differentiation and survival of multipotent mammary stem cells [30]. STAT3 and STAT5 expression can be found in all breast cancer subtypes and a down-regulation of both by different drugs was associated with reduced growth in breast cancer subtypes [31]. In our model, tamoxifen did not significantly affect the activation of STAT3 and STAT5 pathways compared to the control condition (Fig. 4g, h). Thus, these pathways did not appear to be involved in the cytotoxic activity of tamoxifen on EO771 cells.

Chen et al. have shown that downregulation of CREB was associated with inhibition of mammary tumour cell growth by a mechanism that appears to be independent of ER as acting on both triple negative cells (MDA-MB-231) and ER + cells (MCF-7) [32]. Knowing that this pathway is found activated during resistance to tamoxifen [33], the effects of this drug on CREB pathway were investigated in EO771 cells. Interestingly, this pathway was found significantly increased during treatment with tamoxifen (Fig. 4i).

Finally, the potential effect of tamoxifen on metastatic dissemination of EO771 cell was investigated. For this, the production of MMP3, which might be involved in metastatic dissemination of breast cancer [9], was evaluated. An increase in MMP3 production in tamoxifen-treated EO771 cells was observed, compared to untreated cells (Fig. 4j). Thus, despite the cytotoxic activity of tamoxifen on EO771 cells, this treatment could promote metastatic spread due to increased production of MMP3.

**Fig. 5:**
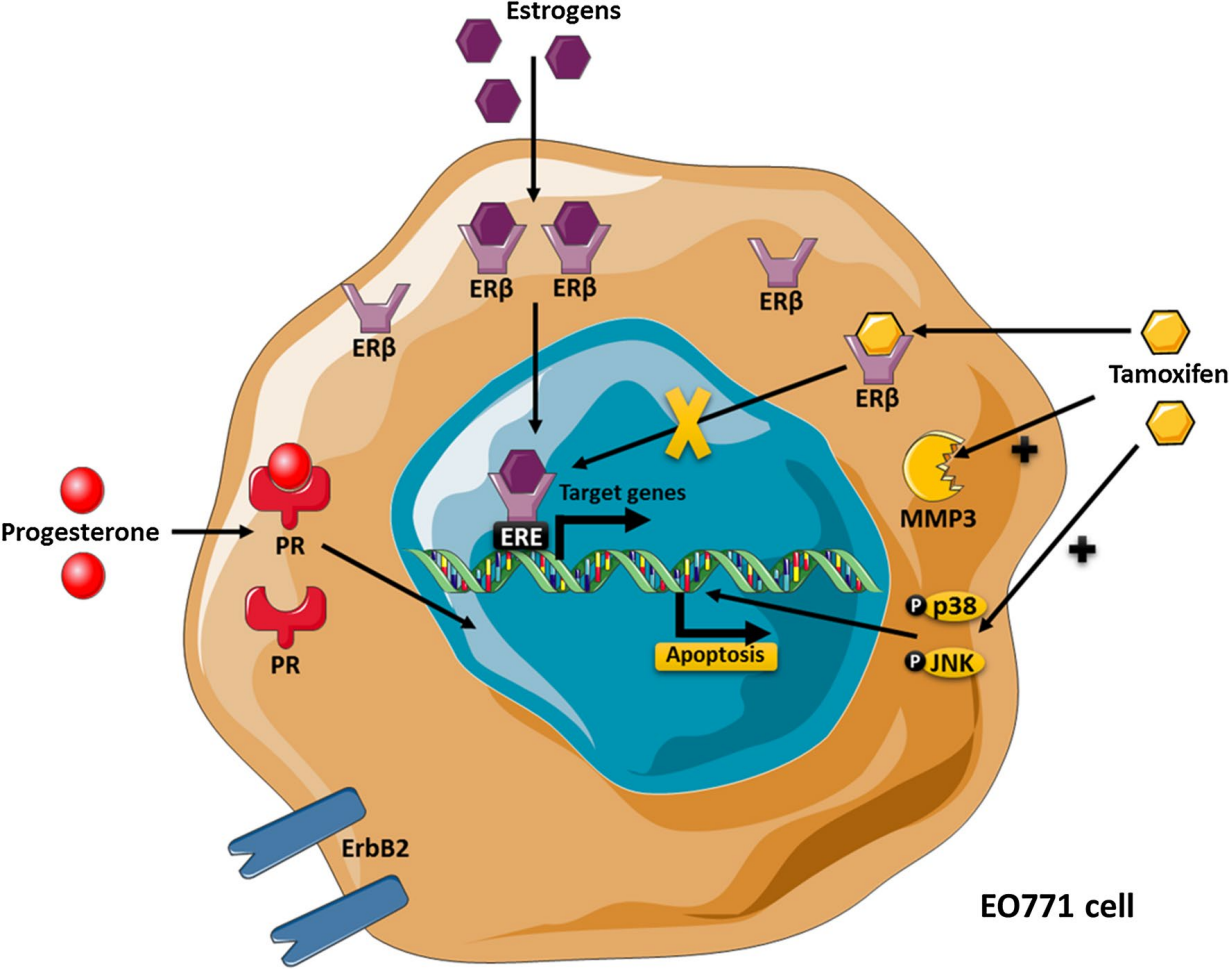
The EO771 phenotype and their sensitivity to tamoxifen. EO771 cells express ERβ, PR and HER2. In presence of estrogens, ERβ are translocated to the nucleus where they bind to the estrogen response elements (ERE) inducing transduction of target genes. Anti-estrogen treatments, such as tamoxifen, block this effect. On the contrary, tamoxifen induces the activation of p38 and JNK pathway, leading to apoptosis, but also increases the MMP3 level

In summary, EO771 presented a luminal B phenotype with the expression of ERβ, PR and ErbB2. They were sensitive to tamoxifen, which induced activation of pro-apoptosis pathways, such as p38 and JNK but also an increased MMP3 level (Fig. 5).

## Discussion

The need to find mouse models to mimic breast cancer is essential to improve the management of this cancer, which remains the deadliest in women. EO771 cells, derived from a spontaneous tumour of C57BL/6 mice [34], are used in syngeneic models [8, 25]. This model, although frequently used, remained poorly characterized. At the start of this study, the aim was to confront the 2 hypotheses which were opposed in literature for this cell line: either the EO771 cells were of the triple negative subtype, or they expressed the ERα (suggesting a luminal A subtype). Thus, our aim was to compare them to a triple negative breast cell line (MDA-MB-231) or luminal A subtype (MCF-7). At the final endpoint of this study, taking account all the results, we concluded that the EO771 cells were a luminal B subtype. This work shows that EO771 cells expressed hormonal receptors, and more particularly ERβ. The phenotype study of these cells also showed that they expressed ERBB2 more than MCF-7 cells. Thus, these cells exhibited the characteristics of luminal B subtype [5], ER + , PR + and ERBB2 +. According to the literature [35], a lower expression for ERα and PR was observed compared to luminal A cells (MCF-7). These frequent tumours are found in about 30 to 40% of breast cancers [35, 36] and are generally more aggressive and high grade, with a worse prognosis than luminal A breast cancers [5]. As in patients with luminal B tumour, EO771 cells were sensitive to tamoxifen therapy suggesting that ERβ would mediate its anti-tumour activity, whereas it is generally associated with ERα. This line could also be derived from breast cancer initiating cells because Ma et al. have reported an absence of ERα but an upregulation of ERβ in breast cancer cells with tumour-initiating capabilities with phenotypic stem cell markers [37]. Thus, knowing now the phenotype of this line EO771, the latter can be used to test many anti-tumour molecules [5] such as selective inhibitors of ERβ [38].

Anti-estrogens are part of the molecules conventionally used to treat luminal cancers. Anti-estrogenic molecules are used in mouse models to assess their effectiveness, alone or in combination [39, 40]. There are several murine mammary tumour cell lines. The most described are those from mice of BALB/c strains such as the 4T1 or TS/4 lines [41]. However, the C57BL/6 mouse is the most widely used inbred strain and the first to have genome sequenced [6]. This strain is a permissive background for maximal expression of most mutations. However, despite this massive use, few breast tumour lines result from C57BL/6 genetic background. Indeed, in addition to EO771 cells, only 9 lines are derived from C57BL/6 mice: 34T, AT-3, M158, MG1361, MMT060562, Py230, Py8119, WT145 and WT276. However, none of the lines mentioned above have a phenotype characterization allowing them to be classified among the luminal subtype which nevertheless represents the majority of breast cancers encountered in patients. Thus, this publication makes it possible for the first time to characterize a murine mammary tumour cell line belonging to the luminal B subtype and derived from the strain C57BL/6.

Among anti-estrogens, tamoxifen is the most used, inducing in these cells the activation of pro-apoptotic pathways involving JNK and p38-MAPK. These latter exert an anti-tumoral effect which is of interest when treating this type of tumour. However, the use of this drug was also associated with an increase in MMP3, known to possess pro-metastatic activity, and of CREB, known to be associated with treatment resistance. Indeed, this MMP3 was found to be increased in EO771.LMB cells, isolated from a spontaneous lung metastasis from an EO771 tumour-bearing compared with parental EO771 [9]. It would be interesting to see if treatment with tamoxifen would fail to induce an increase in metastatic spread in a mouse model with EO771 mammary tumours in agreement with a luminal subtype B, known to be more aggressive.

## Conclusion

Thus, the phenotyping of the EO771 line classified this line in the luminal subtype B allowing for a parallel between the results of the in vitro and in vivo studies, obtained with this murine model, and luminal B breast cancers encountered in patients. This EO771 cell line corresponds to one of the subtypes most frequently encountered in patients and which is associated with a poor prognosis.

## Supplementary information

**Additional file 1: Figure S1** The GAPDH mRNA levels are consistent across EO771, MCF7, and MDA-MB-231 cell lines. **Figure S2** EO771 cells weakly express aromatase mRNA.

## Data Availability

The datasets used and/or analysed during the current study are available from the corresponding author on reasonable request.

## Abbreviations

4-OH-tamoxifen: 4-Hydroxy-tamoxifen
ATCC: American Type Culture Collection
CREB: CAMP-response element binding protein
DMSO: Dimethyl sulfoxide
E2: Estradiol
ERα: Estrogen receptor alpha
ERβ: Estrogen receptor beta
ERBB2: Erb-B2 Receptor Tyrosine Kinase 2
ERK: Extracellular signal-regulated kinase
GAPDH: Glyceraldehyde-3-phosphate dehydrogenase
HER2: Human epidermal growth factor receptor 2
IC50: Concentrations inhibiting 50% of cell viability
JNK: C-Jun NH2-terminal
MAPK: Mitogen-activated protein kinase
MMP3: Matrix metalloproteinase-3
NF-κB: Nuclear factor-κB
PBS: Phosphate-buffered saline
PI3K: Phosphoinositide 3 kinase
PR: Progesterone receptors
q-PCR: Quantitative real-time Polymerase Chain Reaction
STAT: Signal Transducer and Activator of Transcription
