# Supplementary material for: EO771, the first luminal B mammary cancer cell line from C57BL/6 mice
Source: Cancer Cell Int. 2020 Jul 20;20:328. doi: 10.1186/s12935-020-01418-1 (PMC7372867; doi:10.1186/s12935-020-01418-1)
Supplement: Supplementary file 1 — Additional file 1: Figure S1 The GAPDH mRNA levels are consistent across EO771, MCF7, and MDA-MB-231 cell lines. Figure S2 EO771 cells weakly express aromatase mRNA. [file 12935_2020_1418_MOESM1_ESM.docx]

*Supplementary Figure 1: The GAPDH mRNA levels are consistent across EO771, MCF7, and MDA-MB-231 cell lines*

The relative mRNA levels coding for GAPDH were evaluated on MCF-7, MDA-MB-231 and EO771 cells. Relative quantification was obtained by comparing their Ct. P values of <0.05 (*) using a Wilcoxon-Mann Whitney test indicate a significant difference.

*Supplementary Figure 2: EO771 cells weakly express aromatase mRNA*


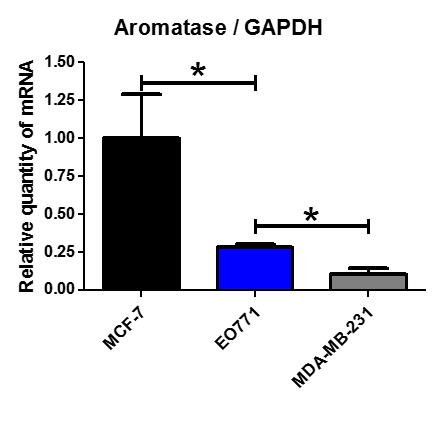


The relative expression of mRNA coding for aromatase was evaluated on MCF-7, MDA-MB-231 and EO771 cells. The values are normalized to the GAPDH gene expression. The data from MCF-7 were set to 1 and the relative quantity of mRNA is shown. P values ​​of <0.05 (*) using a Wilcoxon-Mann Whitney test indicate a significant difference.
